# Effects of Post-awakening Light Exposure on Heart Rate Variability in Healthy Male Individuals

**DOI:** 10.1007/s10484-023-09581-7

**Published:** 2023-03-27

**Authors:** Katja Petrowski, Liza Mekschrat, Stefan Bührer, Martin Siepmann, Christian Albus, Bjarne Schmalbach

**Affiliations:** 1 Medical Psychology & Medical Sociology, University Medical Center of the Johannes Gutenberg - University Mainz, University Medicine Mainz, Duesbergweg 6, 55128 Mainz, Germany; 2 Department of Psychotherapy and Psychosomatic Medicine, University Hospital Carl Gustav Carus, Technische Universität Dresden, Dresden, Germany; 3 Department of Psychosomatic Medicine and Psychotherapy, University Medical Center of University Cologne, Cologne, Germany

**Keywords:** LED light-effects, Heart rate variability (HRV), Retinal-Ganglion-Cells (RGCs), Morning, hypothalamic suprachiasmatic nucleus (SCN)

## Abstract

Light-induced effects on the autonomic nervous system (ANS) are assumed to be mediated by retinal projections to the hypothalamic suprachiasmatic nucleus (SCN) via different routes. Light information for the circadian system is detected by a subset of intrinsically photosensitive retinal ganglion cells (ipRGCs), however, inconsistency exists in research concerning the effects of light exposure on heart rate variability (HRV). Two within-subject experiments were conducted in a standardized sleep laboratory to investigate effects of light intensity (study I, *n* = 29: 2 days dim vs. bright light) and spectral composition (study II, *n* = 24: 3 days using red vs. blue vs. green light) on HRV parameters (RMSSD, LF, HF-HRV, LF/HF ratio). Light exposure was conducted for one-hour in the post-awakening phase at 5:00 AM. Results revealed no significant light intensity effect comparing dim light versus bright white light on HRV parameters. Light color of different wavelengths significantly influenced all HRV parameters except the low frequency, with moderate to large effect sizes. RMSSD values were elevated for all three colors compared to norm values, indicating stronger parasympathetic activation. LED light of different spectral compositions demonstrated bidirectional effects on spectral components of the HRV. Red light decreased the LF/HF ratio within 30 min, whereas with blue light, LF/HF ratio consistently increased across 40 min of light exposure.

## Introduction

Light-induced effects on the autonomic system are assumed to be mediated by retinal projections to the body’s central circadian pacemaker, the hypothalamic suprachiasmatic nucleus (SCN), via different routes (Dickmeis, 2009; Ishida et al., 2005; Ulrich-Lai et al., 2006). Light information for this circadian system is detected by a subset of melanopsin-expressing retinal ganglion cells (mRGCs), also referred to as intrinsically photosensitive retinal ganglion cells (ipRGCs), with a characteristic spectral sensitivity pattern (Berson et al., 2002; Brainard et al., 2001; Figueiro et al., 2018; Hattar, 2002; Hattar et al., 2003; Provencio et al., 2002). Peak sensitivity is at ~ 480 nm, i.e., in the short-wavelength (blue) light spectrum (Al Enezi et al., 2011; Berson et al., 2002; Dacey et al., 2005; Hankins et al., 2008). Light exposures using this peak sensitivity wavelength or bright light of broader wavelengths have stimulatory effects on the hypothalamic–pituitary–adrenal (HPA) axis activity (Hatanaka et al., 2008; Ishida et al., 2005; Niijima et al., 1992). Furthermore, the SCN affects the autonomic nervous system (ANS; Black et al., 2019), specifically body temperature, alertness, and attention (Cajochen et al., 2005; Litscher et al., 2013). This effect can be reflected in heart rate variability parameters (HRV; Edelhäuser et al., 2013; Scheer et al., 1999).

Concerning the general effect of light exposure on ANS, findings have shown that light exposure (700 lx, fluorescent light) suppresses autonomous vagus functions without affecting the respiratory system (9:00 PM, 10 min; Schäfer & Kratky, 2006). Bright light exposure (above 5000 lx; LED) leads to a significant increase in heart rate (HR) by raising sympathetic activity of the autonomic nervous system (12:00–4:00 PM, 4 h; Rüger et al., 2006).

Results of studies applying different spectral distributions (visual colors) of light exposure are, however, quite diverse. In one study, a significant decrease in the heart rate (HR) following blue light exposure was observed, whereas HR was not significantly altered after exposure to red light (9:00–11:00 AM, 10 min, 140 lx; Litscher et al., 2013). With monochromatic blue light exposure over a long period of 120 min however, heart rate (HR) decreased at a slower rate compared to green light exposure (11:00 PM, 120 min, Cajochen et al., 2005).

Concerning HRV parameters, blue fluorescent light did not increase the high frequency (HF-HRV) significantly (9:00 PM, 10 min, 700 lx; Schäfer & Kratky, 2006). Exposure to red light using colored glass panels with daylight significantly increased the low frequency (LF) oscillations without any effects for other colors (10:00 AM–1:00 PM 10 min, 50 lx; Edelhäuser et al., 2013). Furthermore, the LF/HF ratio during red light (631 nm, 140.98 lx) exposure significantly increased over time indicating rising sympathetic activity (Litscher et al., 2013). This might be explained by red light-accelerated blood circulation and stimulation of the sympathetic nervous system compared to green and blue light (Lee et al., 2018).

These inconsistent findings for the different colors of light in previous studies might be explained by various durations of light exposure, differences in spectral distributions, small sample sizes as well as differences in the time of day. Thus, the current study attempted to clarify those fundamental research inconsistencies concerning the effects of standardized colored LED light exposure on HRV metrics (measures of cardiac autonomic modulation), specifically in the post-awakening phase. The post-awakening phase was chosen in order to control light exposure before start of the experimental condition. Two independent within-subject experimental sleep laboratory studies examined the principal influence of 1 h post-awakening light. Study I compared bright white light vs. dim light exposure on HRV, aiming for replication of the findings in the literature. In contrast, study II compared the different effects of blue, red, and green light exposure on HRV.

Taking the results of the study conducted by Rüger et al. (2006) on day-time and night-time light exposure into account, it can be hypothesized that bright white light (similar to daylight) increases HR when compared to dim light. Based on the results of the study conducted by Cajochen et al. (2005) a higher HR and lower HRV for blue light compared to red light can be assumed.

## Method

### Participants

Two samples of *N* = 29 (study I) and *N* = 24 (study II) healthy adults were included. Descriptive sociodemographic information on the included participants is provided in Table 1. The participants were recruited and tested between December 2016 and May 2017. Recruitment took place via online advertisement. Study participation was compensated with 50 euros per night, with either 2 nights in study 1 or 3 nights in study 2. Prior to participation, the volunteers were screened for potential exclusion criteria by telephone using a standardized screening protocol, which included psychological disorders, acute and chronic illnesses, such as auto-immune diseases, coronary heart disease, disorders with chronic inflammation, metabolic disorders, blood disorders, or allergies. These were chosen because they constitute factors known or suspected to influence the ANS. Volunteers were also excluded if they reported any use of psychoactive drugs, smoking of more than ten cigarettes per day and a BMI of > 27 kg/m. Furthermore, only participants between the ages of 18 and 35 were recruited for the study to circumvent the potential impact any age-related eye-diseases (such as age-related macular degeneration) might have on the effectiveness or functionality of light exposure. Data collection took place as part of a larger project within one of six data collection sub-projects. A previously published article by the authors focused on the cortisol awakening response after light exposure (see Petrowski et al., 2019). The study protocol was approved by the Ethics Committee of the Medical Faculty of the Technical University of Dresden, Germany (No #EK353092014) and was conducted in accordance with the Declaration of Helsinki (1964). All the participants provided written informed consent prior the laboratory testing.

**Table 1:** Sociodemographic information for both samples

	Study 1 (*n* = 29)	Study 2 (*n* = 24)	Comparison
Age	24.08 (3.12)	22.83 (3.33)	*t*(51) = 1.41, *p* = .165, *d* = 0.39 [− 0.159; 0.933]
Gender			*χ*^2^(1) = 0.02, *p* = .895
Female	0	0	
Male	29	24	
Family status			*χ*^2^(4) = 6.94, *p* = .139
Single	12	10	
Committed relationship	11	13	
Married	1	0	
Separated	0	1	
Missing	5	0	
Education			*χ*^2^(2) = 2.36, *p* = .308
≤ 9 years	0	1	
10 years	0	1	
≥ 12 years	26	21	
Training qualification			*χ*^2^(5) = 6.10, *p* = .297
Still training	12	13	
Completed apprenticeship	2	2	
University	6	3	
No training completed	3	4	
Other	1	2	
Missing	5	0	

### Procedure

Both studies applied within-subject experimental designs and identical sleep laboratory settings. The independent variables were two light conditions (bright vs. dim) in *study I* and three light conditions (red vs. blue vs. green) in *study II.* The order of the light conditions was counterbalanced and randomized in both studies. HR and HRV were collected as central biological outcome measures across the post-awakening period in both studies.

Testing took place at the university’s sleep laboratory. The participants were advised via e-mail to refrain from alcohol and any strenuous physical activity or exercise on the days prior to the testing nights and on subsequent mornings. Participants completed questionnaires, were tested for color vision, fitted with the motion sensor, and introduced to the testing procedure of the following day after their arrival. Participants went to bed in a room with fully darkened windows at 11 PM. As pre-announced, the participants were awakened by the study team at 5:00 AM the next morning. This relatively early wake-up time corresponds to the methodology of an early work schedule (e.g. Figueiro & Rea, 2012) and was chosen in the present study to reduce the likelihood of participants waking up prior to the pre-specified time. Sleep quality as well as earlier wake-up were monitored by actigraphy. The participants were instructed to wear dark sunglasses (< 2 lx) when using the restrooms during the night and after wake-up.

In the 5 min after wake-up, participants were allowed a visit to the restroom and the study team equipped them with the HRV tools as described below. The experimental light exposure started at 5:05am after awakening. During the 60 min of light exposure, four equal time-sequences were chosen and markers were set to allow an accurate post-processing of data. The first sequence was the baseline for both studies. The testing took place on consecutive days during 1 week.

### Light Exposure

Two half Ulbricht spheres were used to generate LED light, with the bulbs being positioned on the inside around the opening for a consistent illumination. The LEDs were covered with a spectral selective diffusor to ensure a homogeneous illumination of the participants’ retina. Participants of both studies were positioned in a chair in front of the light sources with their chin resting on a chinrest so that their faces reached into one half-sphere (2PI-Geometry) and the eye-level illumination was the same for each participant. The LEDs were controlled remotely via computer (USB to DMX Controller) and powered by electrical DC-dimming. Across the two experiments, four different light exposure settings were used, producing white (bright and dim), blue, red, and green light. The light exposure consisted of the following lighting conditions: narrow-band LEDs, blue (201 lx; peak wavelength 470–480 nm); green (806 lx; peak wavelength 520 nm); red (235 lx; peak wavelength 635 nm); bright white light (414 lx; mix of blue, green and red light each one third of their corresponding setting); dim white light (< 2 lx) almost dark. Table 2 shows the light settings and specificities that were used as well as the luminance in the sphere and the illuminance at eye level. The four light conditions (bright white, blue, green, and red) were set up in such a way that each condition used the same number of photons, resulting in the previously mentioned illuminances at the subject’s eye level. Standardization by photon density, instead of standardization by illuminance (lux), was chosen since the present study aimed to target the non-visual circadian system/mRGCs rather than the visual system. Hence, in order to examine whether mRGCs respond in various ways to light of different wavelengths and thus exert a difference influence on HRV, it was considered critical to standardize light conditions by the entity that actually mediates the photo-biological response at the receptor level, photon density in this instance (Brainard et al., 2001). This variance from luminance perception, a phenomenon dependent on the spectral sensitivity of the visual system, was not the focus of the present study (see Table 2).

**Table 2:** Light exposure characteristics

		Red	Green	Blue	White 1/3 R + G + B
Number of photons		4.26E + 14	4.26E + 14	4.26E + 14	4.26E + 14
Spectral irradiance	W/m^2^	1.341	1.598	1.760	1.566
Illuminance at the eye	Lux	235	806	201	414
(Narrow band) Spectra with different peak wavelength	nm	635	520	475	Combination of RGB
Luminance within the half Ulbricht sphere [cd/sqm]	cd/m^2^	74.8	256.6	64.0	131.8

*R* + *G* + *B* mix of red, green, and blue

Exposure to light took place in a darkened room with stray light levels below < 1 lx (at the eye level). Intensity of illuminance (lux) was controlled at eye level before and after light exposure each day using an illumination meter (Pocket Lux 2; Lichtmesstechnik GmbH Berlin, Germany).

### Heart Rate Variability

The appliances for measuring HRV consisted of Polar RS-800CX® watches, wireless RSCX800 Science® sensory belts with integrated electrodes, and Polar Pro Trainer 5 (version 5.40.170). Participants were instructed to assume a comfortable sitting position during measurement. Following guidelines of current recommendations (Laborde et al., 2017) and defined standards carried out by the Task Force of The European Society of Cardiology and the North American Society for Pacing and Electrophysiology (Malik et al., 1996), for 5-min time phases were chosen for analyses. Those interference-free phases were set between 5–10 min, 25–30 min, 35–40 min and 55–60 min after light onset. The time domain variable RMSSD measures short-term fluctuations in milliseconds (ms) and reflects parasympathetic nervous system (PNS) activity. For the spectral analysis of certain frequency bands, the frequency domain variables low frequency (LF: ranging from 0.04 to 0.15 Hz), high frequency (HF: ranging from 0.15 to 0.40 Hz) as well as its ratio in percent ((LF/HF)*100) were applied. The raw files (HR) were corrected with the Polar ProTrainer 5 filter (filter power: moderate, minimum protection zone: 6 sqm) and manually checked for correct beat-to-beat RR interval detection (for instance without ectopic beats or erroneous values of the RR interval) in the post-processing of recordings. A trained inspector extracted values from these recording phases and saved them to import data into SPSS Statistics v.26 (IBM, Chicago, IL, USA).

### Statistical Analysis

All statistical analyses were carried out with SPSS for Windows, version 26 (IBM, Chicago, Illinois). First, HRV outliers of more than three standard deviations above or below the mean of each sampling point were removed and subsequently replaced by multiple imputation together with any missing data. For the analyses, we calculated a 2 (light condition: dim, bright white) × 4 (measurement points, see procedure) repeated-measures ANOVA for Study I and a 3 (light condition: red, blue, green) × 4 (measurement points) repeated-measures ANOVA for Study II. If the assumption of sphericity was violated, we applied the Greenhouse–Geisser correction. Since group sizes were almost identical across conditions, no further corrections were applied. Each ANOVA was performed for the dependent variables (DVs) RMSSD, LF-HRV, HF-HRV, and LF/HF ratio. In addition to partial *η*^2^_*p*_ effect size estimates we also report the associated 90% confidence intervals. All figures were created in Microsoft Excel 2019.

## Results

### Study I: Bright vs. Dim Light

**Fig. 1:**
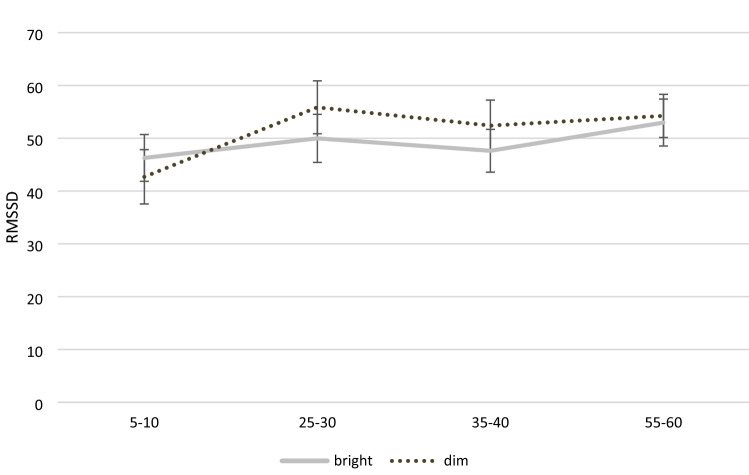
Mean (± *SE*) RMSSD across measurement points for bright and dim light conditions

**Fig. 2:**
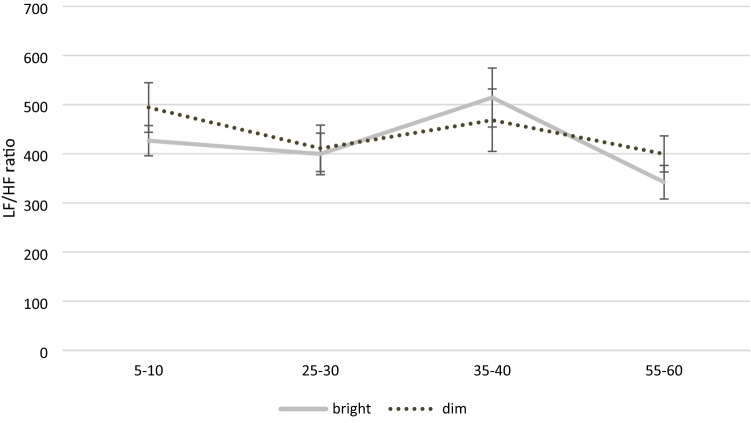
Mean (± *SE*) LF/HF ratio across measurement points for bright and dim light conditions. Time intervals between 5 and 10 min, 25 and 30 min, 35 and 40 min, and 55 and 60 min

First, paired sample *t*-tests were conducted to compare the baseline parameters for the four DVs, finding no significant differences between bright and dim light for any of the DVs, all *t-values* ≤ 1.29, *p-values* ≥ 0.207, Cohen’s *d-values* ≤ 0.24 (see Table 3 for descriptive statistics). As shown in Table 4, none of the main or interaction effects of bright versus dim light were significant, with the highest effect size being *η*^2^_*p*_ = 0.056. However, time had a substantial impact on all of the DVs, with *η*^2^_*p*_ ranging between 0.080 and 0.140 and all but one of the effects being significant (see Figs. 1 and 2).

**Table 3:** Means and standard deviations for all dependent variables as well as heart rate across measurement points by light condition

Light condition					
Study I			Study II		
	Bright	Dim	Red	Blue	Green
HR			HR		
1	79.55 (28.51)	71.10 (19.34)	78.77 (29.84)	79.09 (24.38)	87.87 (30.40)
2	66.60 (8.65)	66.16 (9.08)	63.91 (8.86)	67.30 (9.24)	65.47 (10.27)
3	66.21 (8.01)	68.43 (12.11)	64.85 (7.75)	67.11 (10.03)	66.10 (10.51)
4	70.17 (14.22)	69.67 (10.91)	65.05 (9.52)	69.09 (8.84)	68.97 (9.64)
RMSSD			RMSSD		
1	46.27^a^ (23.86)	42.69^a^ (27.66)	39.61^a^ (18.08)	36.09^a^ (22.93)	29.99^a^ (23.33)
2	49.97^a^ (24.44)	55.85^a^ (26.97)	53.16^a^ (17.28)	45.33^a^ (21.04)	45.72^a^ (13.09)
3	47.62^a^ (21.84)	52.39^a^ (26.03)	57.35^a^ (20.73)	49.60^a^ (26.06)	49.84^a^ (21.79)
4	52.96^a^ (23.91)	54.23^a^ (22.03)	62.92^a^ (26.29)	48.15^b^ (21.49)	49.93^a, b^ (17.91)
LF			LF		
1	2770.95^a^ (1864.57)	2491.10^a^ (1669.54)	2255.67^a^ (1201.33)	2162.26^a^ (1666.47)	1870.31^a^ (1875.04)
2	2467.96^a^ (1393.61)	2899.27^a^ (1562.43)	3036.21^a^ (1331.09)	3128.78^a^ (1945.59)	2736.70^a^ (1239.45)
3	2897.06^a^ (1714.29)	2978.5 ^a^ (1812.44)	3446.17^a^ (1218.67)	2772.21 ^a^ (1340.89)	3106.24^a^ (1674.98)
4	3112.86^a^ (1885.58)	3165.64^a^ (1332.55)	4050.94^a^ (1978.53)	2713.32 ^b^ (1550.55)	3563.73^a, b^ (1771.37)
HF			HF		
1	873.31^a^ (998.30)	812.29 ^a^ (993.02)	591.37 ^a^ (423.00)	562.35 ^a, b^ (655.32)	293.76^b^ (280.62)
2	1008.62^a^ (1095.18)	1277.21^a^ (1275.83)	1020.12^a^ (679.97)	756.11^a, b^ (794.05)	634.88^b^ (323.50)
3	855.85^a^ (837.74)	980.99^a^ (893.65)	990.94^a^ (573.62)	1023.60^a^ (1409.25)	728.39^a^ (517.80)
4	1120.92^a^ (807.69)	1078.38^a^ (862.16)	1130.87^a^ (786.36)	738.27^a^ (548.67)	709.16^a^ (393.91)
LF/HF ratio			LF/HF ratio		
1	426.65^a^ (166.22)	494.18^a^ (271.13)	517.57^a^ (198.07)	523.32^a^ (245.71)	641.35^a^ (469.59)
2	399.62^a^ (227.67)	411.09^a^ (254.66)	417.98^a^ (247.49)	571.36^a^ (340.74)	483.67^a^ (208.60)
3	514.48^a^ (322.64)	468.37^a^ (341.90)	414.91^a^ (244.28)	612.30^b^ (398.65)	546.04^a, b^ (322.95)
4	342.16^a^ (184.27)	399.64^a^ (197.61)	387.35^a^ (145.43)	455.78^a, b^ (274.21)	514.11^b^ (193.14)

Standard deviations in parentheses

Means with different superscripts [a, b] differ at *p* < .05 (Bonferroni-corrected) between light conditions

**Table 4:** Analyses of variance for all dependent variables as well as HR across measurement points by light condition

Study I (bright vs. dim light)			
	Intensity	Time	Intensity × time
RMSSD	*F*(1, 28) = 0.25, *p* = .623, η^2^_*p*_ = .009, [0; .128]	*F*(1.89, 52.92) = 4.55, *p* = .005, η^2^_*p*_ = .140, [.015; .267]	*F*(3, 84) = 1.39, *p* = .252, η^2^_*p*_ = .047, [0; .111]
LF	*F*(1, 28) = 0.07, *p* = .794, η^2^_*p*_ = .002, [0; .088]	*F*(2.34, 65.50) = 2.44, *p* = .086, η^2^_*p*_ = .080, [0; .176]	*F*(3, 84) = 1.10, *p* = .353, η^2^_*p*_ = .038, [0; .095]
HF	*F*(1, 28) = 0.20, *p* = .659, η^2^_*p*_ = .007, [0; .121]	*F*(2.38, 66.58) = 3.25, *p* = .037, η^2^_*p*_ = .104, [.003; .206]	*F*(2.42, 67.63) = 1.53, *p* = .222, η^2^_*p*_ = .052, [0; .133]
LF/HF ratio	*F*(1, 28) = 0.39, *p* = .539, η^2^_*p*_ = .014, [0; .144]	*F*(2.06, 57.64) = 3.77, *p* = .028, η^2^_*p*_ = .119, [.007; .235]	*F*(3, 84) = 1.66, *p* = .183, η^2^_*p*_ = .056, [0; .124]
HR	*F*(1, 27) = 0.73, *p* = .401, η^2^_*p*_ = .026, [0, .177]	*F*(1.14, 30.86) = 7.88, *p* < .001, η^2^_*p*_ = .226, [.040, .403]	*F*(1.21, 32.68) = 6.09, *p* = .014, η^2^_*p*_ = .184, [.022, .357]

Study II (red vs. blue vs. green light)			
	Color	Time	Color × time
RMSSD	*F*(2, 46) = 4.35, *p* = .019, η^2^_*p*_ = .159, [.016; .294]	*F*(2.11, 48.54) = 22.65, *p* < .001, η^2^_*p*_ = .496, [.306; .601]	*F*(3.25, 74.70) = 0.78, *p* = .521, η^2^_*p*_ = .033, [0; .084]
LF	*F*(2, 46) = 1.71, *p* = .193, η^2^_*p*_ = .069, [0; .183]	*F*(2.04, 46.83) = 17.99, *p* < .001, η^2^_*p*_ = .439, [.241; .556]	*F*(6, 138) = 2.08, *p* = .086, η^2^_*p*_ = .083, [0; .127]
HF	*F*(2, 46) = 4.77, *p* = .013, η^2^_*p*_ = .172, [.022; .308]	*F*(2.03, 46.75) = 9.67, *p* < .001, η^2^_*p*_ = .296, [.107; .429]	*F*(3.56, 81.98) = 1.03, *p* = .392, η^2^_*p*_ = .043, [0; .096]
LF/HF ratio	*F*(2, 46) = 3.82, *p* = .029, η^2^_*p*_ = .142, [.008; .276]	*F*(1.78, 41.00) = 3.30, *p* = .052, η^2^_*p*_ = .126, [0; .266]	*F*(3.34, 76.84) = 1.63, *p* = .184, η^2^_*p*_ = .066, [0; .137]
HR	*F*(2, 30) = .853, *p* = .436, η^2^_*p*_ = .054, [0; .180]	*F*(1.08, 16.22) = 11.19, *p* = .003, η^2^_*p*_ = .427, [.108; .610]	*F*(1.56, 23.34) = 0.31, *p* = .682, η^2^_*p*_ = .020, [0; .141]

Numbers in square brackets represent the 90% confidence interval of the effect size estimate

### Study II: Red vs. Blue vs. Green Light

**Fig. 3:**
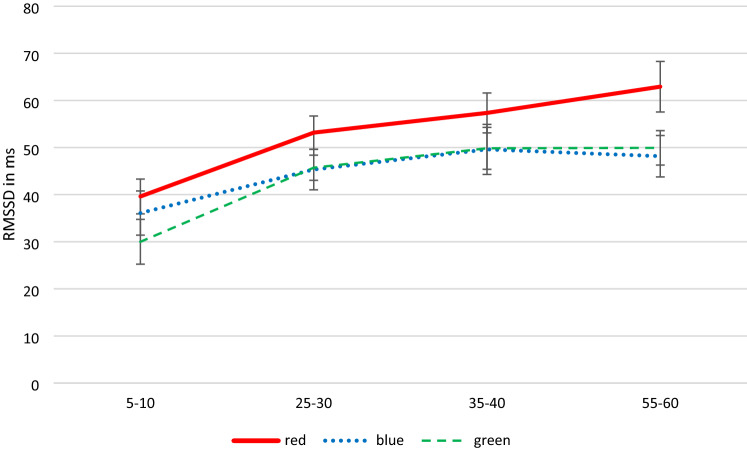
Mean (± *SE*) RMSSD in milliseconds across measurement points for colored light conditions. Time intervals between 5 and 10 min, 25 and 30 min, 35 and 40 min, and 55 and 60 min (Color figure online)

**Fig. 4:**
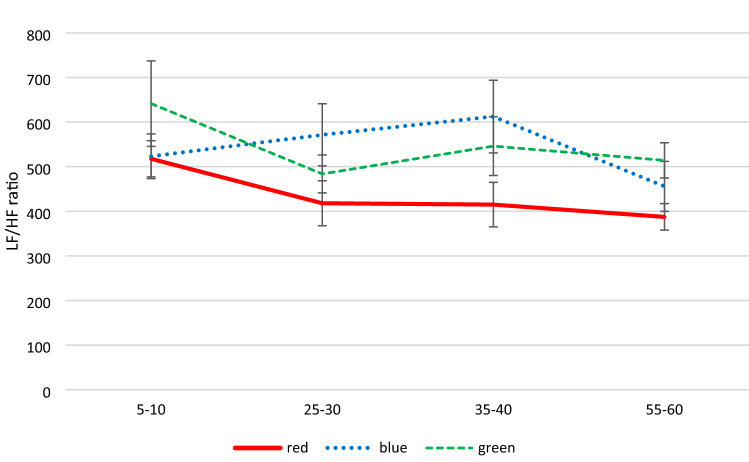
Mean (± *SE*) LF/HF ratio across measurement points for colored light conditions. Time intervals between 5 and 10 min, 25 and 30 min, 35 and 40 min, and 55 and 60 min (Color figure online)

First the baselines for the three light color conditions with regard to the four DVs was compared. The results showed significant differences for the high frequency dependent variable, *F*(2, 46) = 3.71, *p* = 0.032, *η*^2^_*p*_ = 0.139 [0.007; 0.272], with lower values for the green condition (see Table 3). For the other three DVs, baseline levels were comparable, *F*s ≤ 1.43, *p*s ≥ 0.249, *η*^2^_*p*_s ≤ 0.059 (see Table 4). In the main analyses, large effects of time on all of the DVs, with *η*^2^_*p*_ between 0.126 and 0.496 were found. Light color also significantly affected all outcome variables except for LF, with moderate to large effect sizes (*η*^2^_*p*_ between 0.142 and 0.172). The interaction terms were again not significant for any of the DVs (see Figs. 3 and 4).

## Discussion

**Fig. 5:**
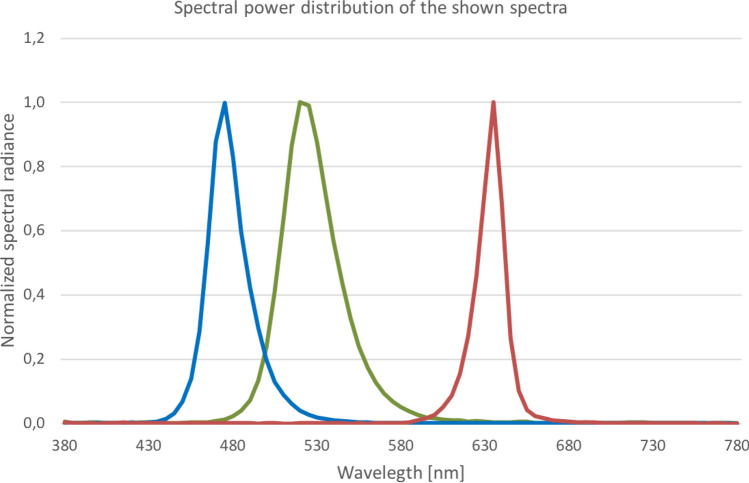
Spectral power distribution of the shown spectra

The effect of light on the autonomic nervous system is mediated by retinal projections to the hypothalamic suprachiasmatic nucleus (SCN; Dickmeis, 2009; Ishida et al., 2005; Ulrich-Lai et al., 2006). A major role of a subset of melanopsin-expressing retinal ganglion cells (mRGCs) with a characteristic spectral sensitivity (see also Fig. 5) pattern has here been detected (Berson et al., 2002; Brainard et al., 2001; Hattar, 2002; Hattar et al., 2003; Provencio et al., 2002). In the present study the stimulatory effect of four different LED light exposures (dim, bright white in study 1; blue, red, and green in study 2) with peak sensitivity wavelengths of bright white (414 lx; mix of blue, green and red light each one third), blue (201 lx; peak wavelength 470–480 nm), green (806 lx; peak wavelength 520 nm) and red (235 lx; peak wavelength 635 nm) on HRV metrics were investigated (Hatanaka et al., 2008; Ishida et al., 2005; Niijima et al., 1992).

First of all, the present results (study 1) revealed no significant main effect of the light conditions dim light versus bright white light for RMSSD, LF, HF-HRV, and LF/HF. This might be in contrast to some literature that found a main effect for bright white light of 100, 800, and 5000 lx and elevated heart rate (Rüger et al., 2006; Scheer et al., 1999). Therefore, the hypotheses cannot be confirmed that bright white light exposure (similar to daylight) leads to an increase in HR and in the LF/HF ratio as well as a decrease in HF-HRV and RMSSD as compared to dim light exposure. Compared to the findings in the literature the present study investigated exclusively post-awakening in order eliminate pre-testing light effects on the system. Furthermore, the age was standardized to circumvent potential age-related eye diseases compared to other studies. In the present study, additionally, a significant difference for time could be identified for RMSSD, HF-HRV and the LF/HF ratio. This is in line with an increasing LF/HF ratio during a daylight exposure previously noted by Edelhäuser et al. (2013). Based on our results time plays not only a crucial role for time domain but also frequency domain HRV parameters when examining LED light (dim versus bright light) and HRV (see Fig. 2).

Concerning the role of the mRGCs and their enervation of the autonomic nervous system a large effect of time on the HRV parameters could be observed. The wavelengths of light colors (study II) significantly influenced all outcome variables except LF over time, with moderate to large effect sizes. After blue light exposure the RMSSD slightly increased and the LF/HF ratio strongly increased whereby after the red-light exposure the RMSSD increased more as well and the LF/HF ratio decreased. Based on the results by Schäfer & Kratky (2006), Cajochen et al. (2005) a greater stimulatory effect on the sympathetic activation at post-awakening by blue LED light exposure was proposed, as reflected by higher LF/HF ratio and lower RMSSD compared to red LED light (H3). The higher LF/HF ratio and the lower RMSSD after blue light exposure compared to red light exposure was replicated in the present study. However, the increase in values of the LF/HF ratio for red light compared to blue light exposure in the findings of the literature (Edelhäuser et al., 2013; Lee et al., 2018; Litscher et al., 2013) could not be confirmed in the present study, but the higher RMSSD compared to blue light.

Regarding the physiological mechanism underlying light exposure on ANS, the intrinsic photosensitive retinal ganglion cells (ipRGC) are known to play a central role for the circadian rhythm (Figueiro et al., 2018) and melanopsin-containing ipRGCs in particular have direct neuronal associations to the SCN as well as non-visual direct and indirect projections to certain brain areas via SCN (Berson et al., 2002). Considering the stronger effect of blue-light exposure on LF/HF and LF when compared to the red wavelength, an effect of the melanopsin-containing ipRGCs could be observed here. Considering the variable LF power of sympathetic and vagal activity, approximately half of the variability proportion in this frequency band is attributable to the PNS with a smaller share of unspecified factors (Shaffer & Ginsberg, 2017). This would be in line with the blue light stimulatory effect on the ipRGCs and the increase in cortisol concentrations (Petrowski et al., 2019, 2021). Additionally, interactions between sympathetic and vagal activity are described as complex, non-linear, and often non-reciprocal (Shaffer & Ginsberg, 2017). A higher LF/HF ratio may reflect sympathetic dominance whereas lower values hint at parasympathetic dominance. This perspective however was challenged, demonstrating that parasympathetic and sympathetic nerve activity contribution of the numerator LF and the dominator HF are highly variable (Billman, 2013), meaning precise physiological underpinnings of the LF/HF ratio still remain unclear and represent a mix of sympathetic and vagal activity (Laborde et al., 2017; Shaffer & Ginsberg, 2017).

Higher RMSSD values after a red-light exposure of the present study could indicate in terms of HRV more parasympathetic activity. Choi et al. (2011) obtained decreased RMSSD values as a result of red-light exposure in small sub samples with anxiety and depression symptoms. As ANS parameters are altered in patients with anxiety and/or depression (Petrowski et al., 2010; Siepmann et al., 2008), clinical implications might even be derived from these particular findings. Taking this study into account, exposure to light of different colors might influence healthy subjects and those with psychological symptoms differently. Comparing colored light exposure during the post-awakening phase between such groups could be of interest to future research and might also narrow down whether subjects with specific psychological symptoms might be provided with therapeutic benefits regarding ANS parameters through exposure to differently-colored light during certain times of day.

In sum, the present results demonstrate that light exposure can alter HRV parameters with different stimulatory effects depending on light color. Overall, the increased RMSSD values for all colors compared to the norm values of the Task Force (Malik et al., 1996) indicate a possibly stronger activation of the PNS and a sufficient recovery of the ANS.

One particular strength of this study is that testing was conducted in the post-awakening phase, which allowed for a standardized LED light exposure setting. Further strengths lie in the consistent illumination at the retina without previous light effects and participants’ stable sitting posture combined with interference-free HRV time phases to compare light exposure effects. The analyses of various HRV parameters which allows for time and frequency domain comparisons to other studies represents another strength. However, there are limitations, affecting primarily the results of the DV HF-HRV. Since there were significant differences between the three colored light conditions at the baseline level, effects for this DV need to be interpreted cautiously, especially for green light. Furthermore, the present investigation is limited by the circumstance that Studies 1 and 2 were not conducted with the same sample, therefore not allowing for within-subject comparisons across both studies. As a comparison of the sociodemographic make-up demonstrates, the participants were similar enough. Future research might be interested in conducting studies as part of which such within-subject comparisons are made possible. Also, it should be noted that the observed statistical power was limited, particularly in the dim vs bright light conditions. Specifically, average power was 0.342 across the dim vs bright analyses, while average power for the colored light analyses was 0.703. While the latter is still below the recommended value, it should be considered acceptable.

In sum, this study was the only one of those introduced in the literature in which the effects of light exposure were measured in the post-awakening phase. While this can be considered a strength of this study. Further research is needed in which researchers take the potential impact of varying chronotypes and sleep quality on HRV or other relevant ANS parameters into account. Furthermore, investigating the interplay of illuminance and wavelength might be a topic of future interest.
